# Visualized peritoneal fluid variation in adolescents and young adults with endometriosis: is there more to it?

**DOI:** 10.3389/frph.2023.1297907

**Published:** 2023-12-15

**Authors:** Abdelrahman Yousif, Mary DePari, Allison F. Vitonis, Holly R. Harris, Amy L. Shafrir, Kathryn L. Terry, Stacey A. Missmer, Naoko Sasamoto

**Affiliations:** ^1^ Department of Obstetrics and Gynecology, Hurley Medical Center, College of Human Medicine, Michigan State University, Flint, MI, United States; ^2^ Department of Obstetrics, Gynecology, and Reproductive Biology, College of Human Medicine, Michigan State University, Grand Rapids, MI, United States; ^3^ Department of Obstetrics and Gynecology, Brigham and Women’s Hospital and Harvard Medical School, Boston, MA, United States; ^4^ Boston Center for Endometriosis, Boston Children’s Hospital and Brigham and Women’s Hospital, Boston, MA, United States; ^5^ Program in Epidemiology, Division of Public Health Sciences, Fred Hutchinson Cancer Center, Seattle, WA, United States; ^6^ Department of Epidemiology, University of Washington, Seattle, WA, United States; ^7^ Division of Adolescent and Young Adult Medicine, Department of Medicine, Boston Children’s Hospital and Harvard Medical School, Boston, MA, United States; ^8^ Department of Nutrition and Public Health, School of Nursing and Health Sciences, Merrimack College, North Andover, MA, United States; ^9^ Department of Epidemiology, Harvard T.H. Chan School of Public Health, Boston, MA, United States

**Keywords:** endometriosis, peritoneal fluid, volume, color, hormones, pelvic pain, adolescents, WERF EPHect

## Abstract

**Background:**

Peritoneal fluid is a medium for endometriosis-associated biomarker discovery from which the local peritoneal environment and pathophysiologic pathways are often inferred. Therefore, we evaluated the associations between peritoneal fluid color and volume at time of endometriosis-related laparoscopic surgery with patient characteristics, endometriosis type and lesion location in adolescents and young adults with endometriosis.

**Methods:**

We conducted a cross-sectional analysis among 545 patients undergoing surgery for endometriosis who enrolled in the Women's Health Study: from Adolescence to Adulthood cohort study. Patient characteristics, surgically visualized endometriosis phenotypes, and gross characteristics of peritoneal fluid were collected in compliance with World Endometriosis Research Foundation Endometriosis Phenome and Biobanking Harmonization Project (EPHect) tools. Chi-square or Fisher's exact tests were applied to test for differences across categories.

**Results:**

Most of the patients were adolescents or young adults (86% age <25 years) of white race (89%), with only superficial peritoneal lesions and rASRM stage = I/II observed at surgery (both 95%). We observed variation in peritoneal fluid color across different menstrual cycle phases at time of surgery (*p* = 0.006). Among those who were cycling at time of surgery, endometriosis patients with red peritoneal fluid were most likely to be in the proliferative phase (49%) compared to the secretory phase (27%), while those with yellow or orange peritoneal fluid were most likely to be in the secretory phase (57% and 86% respectively). Yellow color was significantly less common in those taking combined oral contraceptives but much more common with progesterone only formulation exposure (*p* = 0.002). Peritoneal fluid volume did not differ by cycle phase but was more likely to be low (≤6 ml) for those exposed to hormones at time of surgery (*p* = 0.01). Those with acyclic pelvic pain were less likely to have red peritoneal fluid (*p* = 0.001) but had greater volume (*p* = 0.02) compared to those without.

**Conclusion:**

Our findings highlight the importance of accounting for menstrual cycle phase and hormonal exposures when designing research using peritoneal fluid samples and inferring from biomarker results intended to advance our understanding of endometriosis and associated symptom pathophysiology.

## Introduction

Endometriosis is a chronic inflammatory disorder, often presenting with severe pelvic pain and infertility, that affects approximately 10% of women in their reproductive years globally (1). Endometriosis is characterized by growth of endometrial-like stroma and glands outside the endometrium, with ectopic endometriosis lesions often exhibiting as superficial peritoneal lesions, endometriomas, or deep endometriosis in the pelvic cavity (1).

Peritoneal fluid is a biologic medium within the peritoneal cavity, which can be observed and collected during laparoscopic surgery. While peritoneal fluid is derived by transudation from blood and therefore many substances found in blood can also be detected in the peritoneal fluid (2), the location of peritoneal fluid in the pelvis proximal to ectopic endometriotic lesions makes it a medium that contributes to defining the local environment of the peritoneal cavity. Therefore, peritoneal fluid biomarkers have been studied to understand endometriosis pathophysiology and its biomarkers (3, 4). However, only a few studies have reported the gross characteristics of peritoneal fluid in endometriosis, with inconsistent findings (5–7). There may be many implications for influence of gross characteristics on endometriosis research and biomarker discovery. One pragmatic example is that patients with a higher peritoneal volume at any given surgery could, by definition, have their fluid included more frequently in experiments and observational studies. The objective of this study was to examine the association between gross characteristics of peritoneal fluid (i.e., color and volume) and patient-specific characteristics, including demographics, symptoms, pharmaceutical exposures, menstrual cycle phase, and visualized disease at the time of surgery.

## Materials and methods

### Study population

The Women's Health Study: from Adolescence to Adulthood (A2A) is an observational longitudinal cohort study that enrolled 1,549 pre-menopausal female participants with and without endometriosis [*n* = 787 with surgically-confirmed endometriosis and *n* = 762 without] between November 2012 and June 2018. Details of the study were described previously (8). In brief, participants were recruited from Gynecology and Adolescent Medicine clinics within two tertiary care centers, as well as from the surrounding community via local advertisements, online postings, and word of mouth. All participants were asked to complete an extensive questionnaire at baseline and annual follow-up questionnaires which were expanded from the World Endometriosis Research Foundation Endometriosis Phenome and Biobanking Harmonization Project (WERF EPHect) standard clinical questionnaire, assessing various factors including anthropometrics, behavioral and reproductive characteristics, pain symptom experience, medical history, and medication use (9). Of the 787 surgically-confirmed endometriosis patients enrolled in the A2A study, 608 had surgery at or after enrollment, and 543 had peritoneal fluid collected and had complete surgical forms with both peritoneal fluid color and volume recorded. Additionally, of the 762 participants enrolled without a diagnosis of surgically-confirmed endometriosis, two had a surgery after enrollment where endometriosis was confirmed and a peritoneal fluid sample was collected with complete surgical form data. Thus, this study included 545 surgically-confirmed endometriosis patients with both peritoneal fluid color and volume information at time of their endometriosis-related surgery.

This study was approved by the Boston Children's Hospital (BCH) Institutional Review Board on behalf of both BCH and Brigham and Women's Hospital. Written informed consent was obtained, with both parental consent and participant assent for individuals aged <18 years at enrollment.

### Assessment of surgical phenotype

Each surgery was attended by a research assistant trained specifically for the study who, per dictation and confirmation by the surgeon, completed the WERF EPHect standard surgical form. This form captured endometriosis macro-phenotype (i.e., superficial peritoneal lesion, endometrioma, deep), revised American Society for Reproductive Medicine (rASRM) score, location of endometriotic lesions, appearance (or color) of superficial peritoneal endometriosis lesions, date of last menstrual period, vaginal bleeding on the day of surgery, and medication use in the past 48 h (10). Peritoneal fluid was collected at endometriosis-related surgeries following the WERF EPHect fluids collection standard protocol (11). Deviations from the standard EPHect protocol included that in this study the peritoneal fluid aspirate was housed in an incubator to mimic the body's core temperature until the specimen could be transported to the biorepository for processing. The peritoneal fluid was centrifuged at 300 g for 10 min at 4°C and frozen at −80°C using Mr. Frosty freezing container. The research assistant who received the peritoneal fluid in the laboratory recorded the gross characteristics of the peritoneal fluid samples (i.e., appearance or color and volume) before processing.

### Other variables

Participant characteristics, including body mass index (BMI), race and ethnicity, and pain symptoms [i.e., dysmenorrhea (pain with periods), acyclic/general pelvic pain (pain occurring at times other than with menses), and dyspareunia (pain with vaginal intercourse/penetration)] were abstracted from baseline or annual questionnaires proximal to the endometriosis-related surgeries. BMI was calculated from self-reported weight and height. For women aged 20 years or older, participants were categorized as underweight (BMI < 18.5 kg/m^2^), normal-weight (BMI 18.5–24.9 kg/m^2^), overweight (BMI 25–29.9 kg/m^2)^, or obese (BMI ≥ 30 kg/m^2^) according to World Health Organization (WHO) criteria (12). For participants <20 years, the age- and gender-specific BMI Z-score was calculated, and participants were categorized as underweight (*Z*-score ≤−2), normal weight (*Z*-score >−2 to <1), overweight (*Z*-score 1–2), or obese (*Z*-score >2) (13). Usual severity of pelvic pain with periods or dysmenorrhea was assessed categorically as none, mild (medication never or rarely needed), moderate (medication usually needed), and severe (medication and bed rest needed) among those who self-reported they were cycling in the last 3 months. Any experience of acyclic pelvic pain was assessed in the last 3 months (yes/no), and dyspareunia was assessed by asking if participants aged 18 years or older avoided or interrupted intercourse or penetration in the last 12 months because of pelvic pain (yes/no).

### Statistical methods

We examined the distribution of participant's surgical and clinical characteristics by the peritoneal fluid color (yellow, orange, pink, red) and volume (≤3, 4–6, 7–11, > 11 ml) at the time of their endometriosis-related surgery. For lesion appearance/color type, we created two grouped variables—type A (clear or red lesions) and type B (blue/black, brown, or white lesions), as these groups have been presumed to represent early vs. late lesions. Medications were classified as any antidepressant/antianxiety medication use or any pain medication use (analgesics or anticonvulsants), including both prescription and non-prescription medications. Chi-square or Fisher's exact tests were applied to test for differences across the categories. All statistics were conducted using SAS 9.4 (SAS Institute, Cary, NC).

## Results

Our study population consisted mainly of adolescents and young adults with surgically confirmed with endometriosis (86% were age <25 years old; Table 1), with median age at endometriosis diagnosis being 16-years old. Majority were white race (89%) and were exposed to exogenous hormones at time of their endometriosis-related surgery (83%). The most prevalent hormonal medication used was the combined estrogen + progesterone birth control pills (61%). The majority of patients were found to have only superficial peritoneal lesions (95%) at surgery, which is the most common macro-phenotype of endometriosis (14).

**Table 1:** Characteristics of surgically-confirmed endometriosis cases with gross peritoneal fluid characteristics in the A2A study (*n* = 545).

	*N* (%)
Patient characteristics	
Age at surgery	
≤15	204 (37%)
16–18	161 (30%)
19–24	104 (19%)
≥25	76 (14%)
Race	
Asian	2 (1%)
Black	17 (3%)
White	486 (89%)
Other/unkown^a^	40 (7%)
Non-hispanic ethnicity	452 (93%)
Body mass index^b^	
Underweight	4 (1%)
Normal weight	290 (65%)
Overweight	113 (25%)
Obese	40 (9%)
Hormone use at time of surgery	452 (83%)
Hormone type at time of surgery	
Combined birth control pills	276 (61%)
Progesterone only medication^c^	114 (25%)
Progesterone intrauterine device	17 (4%)
Other, unknown or more than one	45 (10%)
Endometriosis-specific characteristics at time of surgery	
Age at endometriosis diagnosis, median (IQR)	16 (15,19)
Endometriosis macro appearance (visualization) on laparoscopy	
Superficial peritoneal lesion	516 (95%)
Endometrioma	9 (2%)
Deep	14 (3%)
Both endometrioma and deep	2 (0%)
ASRM stage	
Stage I	417 (77%)
Stage II	94 (18%)
Stage III	6 (1%)
Stage IV	22 (4%)

A2A, Adolescence to Adulthood; IQR, interquartile range

^a^ Participants in the Other/Unknown category included multiracial (*n* = 17), other race unknown (*n* = 23).

^b^ For women aged ≥20 years, participants were categorized as underweight (body mass index (BMI) < 18.5 kg/m^2^), normal-weight (BMI 18.5-24.9 kg/m^2^), overweight (BMI 25-29.9 kg/m^2^), or obese (BMI ≥ 30 kg/m^2^) according to World Health Organization (WHO) criteria. For those <20, the age- and gender-specific BMI *z*-score was calculated and participants were categorized as underweight (*z*-score ≤ -2), normal-weight (*z*-score > -2 to < 1), overweight (*z*-score 1–2), or obese (*z*-score >2).

^c^ Progestin only birth control pill (*n* = 54), Birth control progestin injection/shot (*n* = 3), Oral progestins to regulate the cycle (e.g. medroxyprogesterone acetate [Provera], norethindrone acetate [Aygestin] (*n* = 57).

When we examined patient characteristics by peritoneal fluid colors, we did not see clear differences in exogenous hormone exposure status at time of surgery by peritoneal fluid color. However, among those who were on hormones, there were significant differences in the type of hormones used by peritoneal fluid color (*p* = 0.002, Table 2). Among those who were on hormones at surgery, endometriosis patients with orange, pink, or red color peritoneal fluid were more likely to be on combined birth control pills (65%, 62%, and 67% respectively) whereas those with yellow peritoneal fluid was more likely to be taking progesterone only formulation exposure (40%). When we examined pain medications and anti-depressants/anti-anxiety exposures, there were no statistically significant nor informative differences by peritoneal fluid color.

**Table 2:** Patient characteristics, endometriosis type, lesion location, and color by peritoneal fluid color (*n* = 545).

	Peritoneal fluid color				
	Yellow (*n* = 113)	Orange (*n* = 42)	Pink (*n* = 42)	Red (*n* = 348)	*p*-value
Patient and endometriosis-characteristics at time of surgery					
Hormone use at time of surgery					
No	16 (14%)	8 (19%)	7 (17%)	59 (17%)	0.88
Yes	96 (86%)	34 (81%)	34 (83%)	288 (83%)	
Hormone type at time of surgery					
Combined birth control pills	39 (41%)	22 (65%)	21 (62%)	194 (67%)	0.002
Progesterone only medication	38 (40%)	6 (18%)	8 (24%)	62 (22%)	
Progesterone intrauterine device	5 (5%)	2 (6%)	2 (6%)	8 (3%)	
Other hormones	14 (15%)	4 (12%)	3 (9%)	24 (8%)	
Menstrual cycle phase at the time of surgery among 90 participants not using hormonal therapy					
Non-cycling (on hormones)	*N* = 96	*N* = 34	*N* = 34	*N* = 288	
Cycling	*N* = 16	*N* = 8	*N* = 7	*N* = 59	
Proliferative	1 (7%)	0 (0%)	2 (29%)	29 (49%)	0.006
Ovulatory	4 (29%)	0 (0%)	1 (14%)	8 (14%)	
Secretory	8 (57%)	6 (86%)	3 (43%)	16 (27%)	
Irregular cycles	1 (7%)	1 (14%)	1 (14%)	6 (10%)	
Pain medications within 48 h before surgery					
No	88 (78%)	36 (86%)	35 (83%)	282 (81%)	0.69
Yes	25 (22%)	6 (14%)	7 (17%)	66 (19%)	
Anti-depressants/anti-anxiety medications within 48 h before surgery					
No	87 (77%)	36 (86%)	32 (76%)	285 (82%)	0.47
Yes	26 (23%)	6 (14%)	10 (24%)	63 (18%)	
Experiencing vaginal bleeding at time of surgery					
No	94 (96%)	33 (83%)	28 (88%)	214 (66%)	<0.0001
Yes	4 (4%)	7 (18%)	4 (13%)	109 (34%)	
Endometriosis macro appearance (visualization) on laparoscopy					
Superficial only	106 (96%)	42 (100%)	39 (93%)	329 (95%)	0.68
Endometrioma	1 (1%)	0 (0%)	1 (2%)	7 (2%)	
Deep	3 (3%)	0 (0%)	1 (2%)	10 (3%)	
Both endometrioma and deep	0 (0%)	0 (0%)	1 (2%)	1 (0%)	
ASRM stage					
Stage I	87 (80%)	34 (81%)	31 (74%)	266 (77%)	0.16
Stage II	21 (19%)	8 (19%)	8 (19%)	57 (16%)	
Stage III	0 (0%)	0 (0%)	2 (5%)	4 (1%)	
Stage IV	1 (1%)	0 (0%)	1 (2%)	20 (6%)	
Location of superficial endometriosis lesions^a^					
Pelvic Sidewall	80 (73%)	35 (83%)	26 (62%)	264 (76%)	0.12
Uterosacral Ligament	18 (16%)	4 (10%)	5 (12%)	52 (15%)	0.70
Anterior cul-de-sac	57 (52%)	18 (43%)	21 (50%)	196 (56%)	0.32
Posterior cul-de-sac	97 (88%)	40 (95%)	40 (95%)	316 (91%)	0.40
Appearance of superficial endometriosis lesions^b^					
Vascular	40 (36%)	20 (48%)	10 (24%)	102 (29%)	0.04
Clear	103 (94%)	40 (95%)	40 (95%)	333 (96%)	0.69
Yellow	4 (4%)	1 (2%)	3 (7%)	26 (7%)	0.35
Red	87 (79%)	30 (71%)	37 (88%)	311 (90%)	0.001
White	25 (23%)	11 (26%)	15 (36%)	100 (29%)	0.4
Blue/Black	19 (17%)	10 (24%)	8 (19%)	90 (26%)	0.26
Brown	20 (18%)	11 (26%)	11 (26%)	112 (32%)	0.04
Type A lesions (clear or red)	106 (94%)	40 (95%)	42 (100%)	342 (98%)	0.05
Type B lesions (blue/black, brown or white)	50 (44%)	23 (55%)	21 (50%)	210 (60%)	0.02
Pain symptoms^c^					
Dysmenorrhea Severity^d^					
None/Mild	3 (8%)	1 (5%)	1 (5%)	9 (5%)	0.85
Moderate	11 (28%)	4 (18%)	8 (36%)	58 (31%)	
Severe	25 (65%)	17 (77%)	13 (59%)	121 (64%)	
Any acyclic pelvic pain^e^					
No	16 (21%)	11 (31%)	7 (23%)	105 (43%)	0.001
Yes	62 (79%)	25 (69%)	23 (77%)	140 (57%)	
Avoided intercourse or penetration due to dyspareunia^f^					
No	15 (41%)	5 (36%)	1 (12%)	31 (40%)	0.55
Yes	22 (59%)	9 (64%)	7 (88%)	47 (60%)	
Interrupted intercourse or penetration due to dyspareunia^g^					
No	14 (38%)	5 (36%)	2 (40%)	33 (44%)	0.92
Yes	23 (62%)	9 (64%)	3 (60%)	42 (56%)	

Percentages are showing column percentages by the peritoneal fluid colors.

Number of missings for characteristics: hormone use (*n* = 3), hormone type (*n* = 0), menstrual cycle phase among those cycling (*n* = 3), pain medication (*n* = 0), anti-depressants (*n* = 0), vaginal bleeding (*n* = 52), endometriosis type (*n* = 4), ASRM stage (*n* = 5).

^a,b^ Percentage of superficial lesion appearance and location present at the time of surgery.

^c^ Among *n* = 404 who had answered a questionnaire proximal to their surgery (before or after 6 months).

^d^ Usual severity of pelvic pain with periods among subjects who are cycling (n = 281). Number of missing dysmenorrhea severity (*n* = 10).

^e^ Assessed experience in the last 3 months. Number of missing acyclic pelvic pain (*n* = 15).

^f,g^ Assessed in the last 12 months, restricted to *n* = 141 participants age ≥18 who ever had sexual vaginal intercourse or penetration. Number of missing dyspareunia: avoid intercourse *n* = 4, interrupt intercourse, *n* = 10.

We observed differences in menstrual cycle phase at time of laparoscopic surgery by peritoneal fluid color (*p* = 0.006). Among those who were cycling at time of surgery, endometriosis patients with red peritoneal fluid were most like to be in the proliferative phase (49%) while those with yellow or orange peritoneal fluid were most likely to be in the secretory phase (57% and 86% respectively). Consistently, there were significant differences in the patients' vaginal bleeding status at surgery by peritoneal fluid color (*p* < 0.0001), where endometriosis patients with red peritoneal fluid were more likely to be bleeding at time of surgery (34%) and less likely in other colors (4%–18%). We conducted a sensitivity analysis excluding those who reported bleeding at time of surgery (*n* = 124) and those for whom the peritoneal fluid sample was documented to have had gross contamination (*n* = 7), but observed similar results with similar distributions (Supplementary Table S1).

In terms of the endometriosis surgically visualized macro-phenotypes, which 95% of our patients had superficial peritoneal lesions only and rASRM stage I or II, there were no obvious patterns of difference in the endometriosis macro-phenotype or rASRM stage by peritoneal fluid color. However, pink and red color peritoneal fluid were suggestively more likely to be associated with rASRM stage III or IV (*p* = 0.16), although sample size was limited (*n* = 28 with rASRM stage III or IV).

Differences were observed in superficial peritoneal lesion appearance by peritoneal fluid color. Those with pink or red peritoneal fluid were slightly more likely to have any red lesions (88, 90%) than those with yellow or orange peritoneal fluid (79%, 71%; *p* = 0.001). Those with yellow peritoneal fluid were less likely to have type B lesions (i.e., blue/black, brown, or white; 44%) whereas those with other peritoneal fluid colors were more likely to have type B lesions (orange = 55%, pink = 50%, red = 60%; *p* = 0.02).

As for pelvic pains reported prior to surgery, the patients' acyclic pelvic pain experience differed by peritoneal fluid color (*p* = 0.001), with those with red peritoneal fluid less likely to have reported acyclic pelvic pain in the past 3 months (57%) compared to those with the other three colors more likely to report acyclic pelvic pain (69%–79%).

In terms of peritoneal fluid volume and hormone use at time of surgery; patients with low volume (≤3 ml) were more likely to be on hormones (89%) compared to those with the highest volume >11 ml (77%) (*p* = 0.01; Table 3). We did not observe significant differences in type of hormones, menstrual cycle phase, endometriosis macro-phenotype, rASRM stage, or lesion appearance by peritoneal fluid volume. Use of pain medication at time of surgery was suggestively associated with smaller peritoneal fluid volume (23% for ≤6 ml and 13%–16% for >7 ml; *p* = 0.08). There was a significant difference in peritoneal fluid volume by those with and without superficial endometriosis lesions identified in the posterior cul-de-sac (*p* = 0.007), with those having greater peritoneal fluid volume (>11 ml) being more likely to have posterior cul-de-sac lesions (97%) compared to those with less volume (≤3 ml, 88%). When assessing the relationship between peritoneal fluid volume and pain symptoms, there were significant differences in peritoneal fluid volume by acyclic pelvic pain experience (*p* = 0.02). Those with greater volume (>11 ml) were more likely to report acyclic pelvic pain (70%) compared to those with low volume (≤3 ml, 66%), although no linear trend was observed.

**Table 3:** Patient characteristics, endometriosis type, lesion location, and color by peritoneal fluid volume (*n* = 545).

	Peritoneal fluid volume				
	≤ 3ml (*n* = 162)	4–6 ml (*n* = 133)	7–11 ml (*n* = 123)	>11 ml (*n* = 127)	*p*-value
Patient and endometriosis-characteristics at time of surgery					
Hormone use at time of surgery					
No	18 (11%)	17 (13%)	26 (21%)	29 (23%)	0.01
Yes	144 (89%)	116 (87%)	95 (79%)	97 (77%)	
Hormone type at time of surgery					
Combined birth control pills	81 (56%)	72 (62%)	68 (72%)	55 (57%)	0.31
Progesterone only medication	38 (26%)	30 (26%)	17 (18%)	29 (30%)	
Progesterone intrauterine device	5 (3%)	6 (5%)	2 (2%)	4 (4%)	
Other hormones	20 (14%)	8 (7%)	8 (8%)	9 (9%)	
Menstrual cycle phase at the time of surgery among 87 participants not using hormonal therapy					
Non-cycling (on hormones)	N = 144	N = 116	N = 95	N = 97	
Cycling	N = 18	N = 17	N = 26	N = 29	
Proliferative	8 (47%)	5 (31%)	10 (37%)	9 (33%)	0.40
Ovulatory	5 (29%)	2 (13%)	3 (11%)	3 (11%)	
Secretory	2 (12%)	8 (50%)	10 (37%)	13 (48%)	
Irregular cycles	2 (12%)	1 (6%)	4 (15%)	2 (7%)	
Pain medications within 48 h of surgery					
No	125 (77%)	102 (77%)	107 (87%)	107 (84%)	0.08
Yes	37 (23%)	31 (23%)	16 (13%)	20 (16%)	
Anti-depressants/anti-medications within 48 h of surgery					
No	130 (80%)	105 (79%)	102 (83%)	103 (81%)	0.88
Yes	32 (20%)	28 (21%)	21 (17%)	24 (19%)	
Endometriosis macro appearance (visualization) on laparoscopy					
Superficial only	152 (94%)	125 (95%)	118 (96%)	121 (96%)	0.95
Endometrioma	3 (2%)	3 (2%)	1 (1%)	2 (2%)	
Deep	4 (2%)	3 (2%)	4 (3%)	3 (2%)	
Both endometrioma and deep	2 (1%)	0 (0%)	0 (0%)	0 (0%)	
ASRM stage					
Stage I	111 (70%)	104 (79%)	101 (82%)	102 (80%)	0.33
Stage II	35 (22%)	22 (17%)	16 (13%)	21 (17%)	
Stage III	2 (1%)	1 (1%)	2 (2%)	1 (1%)	
Stage IV	11 (7%)	4 (3%)	4 (3%)	3 (2%)	
Location of superficial endometriosis lesions^a^					
Pelvic sidewall	122 (76%)	92 (70%)	93 (76%)	98 (78%)	0.54
Uterosacral ligament	29 (18%)	14 (11%)	13 (11%)	23 (18%)	0.11
Anterior cul-de-sac	87 (54%)	67 (50%)	75 (61%)	63 (50%)	0.30
Posterior cul-de-sac	142 (88%)	113 (85%)	116 (94%)	122 (97%)	0.007
Appearance of superficial endometriosis lesions^b^					
Vascular	60 (37%)	36 (27%)	35 (28%)	41 (32%)	0.26
Clear	151 (94%)	126 (96%)	119 (97%)	120 (95%)	0.65
Yellow	12 (7%)	7 (5%)	9 (7%)	6 (5%)	0.73
Red	133 (83%)	112 (85%)	110 (89%)	110 (87%)	0.40
White	47 (29%)	31 (24%)	39 (32%)	34 (27%)	0.52
Blue/black	34 (21%)	30 (23%)	28 (23%)	35 (28%)	0.60
Brown	44 (27%)	42 (32%)	27 (22%)	41 (33%)	0.21
Any type A lesions (clear or red)	156 (96%)	128 (96%)	122 (99%)	124 (98%)	0.41
Any type B lesions (blue/black, brown or white)	87 (54%)	72 (54%)	67 (54%)	78 (61%)	0.54
Pain symptoms^c^					
Dysmenorrhea severity^d^					
None/mild	3 (5%)	5 (7%)	2 (3%)	4 (6%)	0.05
Moderate	16 (25%)	15 (22%)	33 (46%)	17 (25%)	
Severe	44 (70%)	48 (71%)	37 (51%)	47 (69%)	
Any acyclic pelvic pain^e^					
No	39 (34%)	28 (30%)	45 (49%)	27 (30%)	0.02
Yes	76 (66%)	64 (70%)	47 (51%)	63 (70%)	
Avoided intercourse or penetration due to dyspareunia^f^					
No	19 (31%)	9 (32%)	12 (50%)	12 (50%)	0.21
Yes	42 (69%)	19 (68%)	12 (50%)	12 (50%)	
Interrupted intercourse or penetration due to dyspareunia^g^					
No	20 (36%)	13 (48%)	11 (46%)	10 (42%)	0.68
Yes	36 (64%)	14 (52%)	13 (54%)	14 (58%)	

Percentages are showing column percentages by the peritoneal fluid colors.

Number of missings for characteristics: hormone use (*n* = 3), hormone type (*n* = 0), menstrual cycle phase among those cycling (*n* = 3), pain medication (*n* = 0), anti-depressants (*n* = 0), vaginal bleeding (*n* = 52), endometriosis type (*n* = 4), ASRM stage (*n* = 5).

^a,b^ Percentage of superficial lesion appearance and location present at the time of surgery.

^c^ Among *n* = 404 who had answered a questionnaire proximal to their surgery (before or after 6 months).

^d^ Usual severity of pelvic pain with periods among subjects who are cycling (*n* = 281). Number of missing dysmenorrhea severity (*n* = 10).

^e^ Assessed experience in the last 3 months. Number of missing acyclic pelvic pain (*n* = 15).

^f,g^ Assessed in the last 12 months, restricted to *n* = 141 participants age ≥18 who ever had sexual vaginal intercourse or penetration. Number of missing dyspareunia: avoid intercourse *n* = 4, interrupted intercourse, *n* = 10.

## Discussion

In a sample consisting largely of adolescents and young adults with endometriosis with detailed information on patient characteristics including surgically visualized endometriosis phenotypes, symptoms, and medication exposures, we observed that peritoneal fluid color and volume differed among some characteristics.

Our results showed that menstrual cycle phase and exogenous hormone type used at time of surgery were significantly associated with differences in peritoneal fluid color or volume. Peritoneal fluid is normally present in the pelvic cavity and its volume has been reported to differ corresponding to the menstrual cycle phase in women undergoing laparoscopy (7, 15–17). One study examined peritoneal fluid volume in 158 women who were menstruating and undergoing laparoscopy, and observed an increase in peritoneal fluid volume after ovulation; with 4–9 ml in days 1–13 and 22 ml in day 14–16 of the menstrual cycle (7). This variation in peritoneal fluid volume by the menstrual cycle phase is thought to be due to permeability caused by increasing concentration of estradiol and progesterone (7, 17), although we did not observe clear differences in peritoneal fluid volume by menstrual cycle phase in our study population. Women using hormonal contraceptives (*n* = 40) has been reported to have lower peritoneal fluid volume (average 5.4 ml) compared to women who were menstruating (7), which was consistent with our finding. While we did not observe any significant associations with use of pain medication or antidepressant/anti-anxiety medications and peritoneal fluid color, our data suggested that those who were on pain medication were more likely to have lower peritoneal fluid volume. This could be because pain medications, mainly anti-inflammatory drugs, could lead to reduced inflammation in the pelvis and lower vascular permeability, resulting in lower peritoneal fluid volume.

Our study population consisted primarily of superficial peritoneal only and rASRM stage I or II disease (both 95%). Therefore, we could not infer clear differences by macro-phentype or rASRM stage. While there was no hint of a gradient in volume, we did observe suggestive differences in peritoneal fluid color by rASRM stage, with pink or red colors being more prevalent in stage III or IV diseases. However, we only had 21 endometriosis patients with rASRM stage III or IV and therefore replication in an independent dataset is needed to confirm this finding. Previous studies reported conflicting results on the association between peritoneal fluid volume and rASRM stage of endometriosis. One small study (*n* = 58) examined peritoneal fluid volume in women with endometriosis with different rASRM stage and observed that the average peritoneal fluid volume was lower in those with rASRM stage III or IV endometriosis compared to those with rASRM stage I (7). In contrast, another small study (*n* = 32) reported that those classified as “severe endometriosis” had higher peritoneal fluid volume compared to those classified as “mild or moderate endometriosis” (6). Another study of 272 endometriosis patients, all of whom presented with infertility, reported no correlation between peritoneal fluid color or volume with rASRM stage (5). Further studies are warranted to investigate the association between macro-phenotype and rASRM stage with peritoneal fluid volume.

In our study population, 64% of the patients presented with red peritoneal fluid, which is in line with another study reporting red peritoneal fluid was the most dominant color (88.9%) among patients with endometriosis (5). Red peritoneal fluid likely indicates the presence of blood or hemoglobin. A prior study reported that peritoneal fluid of women with endometriosis has higher concentration of hemoglobin compared to women without, which may suggest a dysfunction of the mechanism responsible for eliminating hemoglobin from the pelvic cavity after retrograde menstruation (18). This study did not observe differences in peritoneal fluid hemoglobin concentration by rASRM stage, although the study was likely to be limited with small sample size (*n* = 110). To our knowledge, the current study is the first to explore the associations between peritoneal fluid color with lesion appearance. Red lesions have been shown to be associated with more active local inflammation (19, 20). It is not clear why we observed those with red lesions were more likely to have red peritoneal fluid, but it is possible that this may be due to having red lesions reflecting more active local inflammation, promoting neovascularization within the peritoneal environment and increased vascular permeability, resulting in microhemorrhage.

Interestingly, patients with acyclic pelvic pain were less likely to present with red peritoneal fluid and more likely to have higher peritoneal fluid volume. In our data, we did not observe differences in prevalence of hormone use or type used at time of surgery by acyclic pelvic pain status, and therefore it is unlikely that hormone medication is driving these results. Interestingly, those with acyclic pelvic pain have greater volume, which could suggest more local inflammation and vascular permeability (17), although more detailed research investigating peritoneal fluid biomarkers will be needed to further elucidate the potential underlying biology.

To our best knowledge, this is the largest study presenting unique data on descriptive, gross characteristics of the peritoneal fluids in predominantly adolescents and young adults with endometriosis. Another strength of this study is that we were able to associate the peritoneal fluid characteristics with detailed surgical phenotype collected at time of endometriosis-related surgery and patient-reported pelvic pain symptoms. Further research examining biomarkers in these peritoneal fluid samples and their relation to color and volume will advance our knowledge of the differences in the local microenvironment. Since our study population is predominantly a young population mainly presenting with pelvic pain and rASRM stage I disease, these results will not be generalizable to endometriosis patients presenting solely with infertility or with higher disease stage. We acknowledge that for some of the patient characteristics, the sample size was small for some categories and therefore warrant caution for interpretation. Future studies with large samples sizes should explore a wider range of endometriosis phenotypic diversity.

In summary, we investigated differences in gross characteristics of peritoneal fluids by patient characteristics and surgically visualized endometriosis phenotypes, highlighting the importance to account for menstrual cycle phase and exogenous hormonal exposures when designing research using peritoneal fluid samples and conducting studies measuring peritoneal fluid biomarkers to advance our understanding of endometriosis pathophysiology.

## Data Availability

Data are not publicly available due to information that could compromise research participants’ privacy and consent. However, experienced scientists who would like to inquire regarding use of data from this study to address specific hypotheses or replicate the analyses in this study may submit an application and research proposal. Data requests must be reviewed and approved by the Brigham and Women's Hospital Institutional Review Broad (https://www.brighamandwomens.org/research/research-administration). All inquiries should be directed to the A2A cohort leadership committee (womenshealthstudy@bwh.harvard.edu). Data sharing will require a fully executed Data Usage Agreement.

## References

[1] Zondervan KT Becker CM Missmer SA. Endometriosis. N Engl J Med. (2020) 382:1244–56. 10.1056/NEJMra1810764 32212520

[2] Hunter RH Cicinelli E Einer-Jensen N. Peritoneal fluid as an unrecognised vector between female reproductive tissues. Acta Obstet Gynecol Scand. (2007) 86:260–5. 10.1080/00016340601155098 17364298

[3] Koninckx PR Ussia A Adamyan L Gomel V Martin DC. Peritoneal fluid progesterone and progesterone resistance in superficial endometriosis lesions. Hum Reprod. (2022) 37:203–11. 10.1093/humrep/deab258 34849906

[4] Ahn SH Singh V Tayade C. Biomarkers in endometriosis: challenges and opportunities. Fertil Steril. (2017) 107:523–32. 10.1016/j.fertnstert.2017.01.009 28189296

[5] Wu HM Tzeng CR Chen CH Chen PH. Pelvic endometriosis with peritoneal fluid reduces pregnancy rates in women undergoing intrauterine insemination. Taiwan J Obstet Gynecol. (2013) 52:512–5. 10.1016/j.tjog.2013.10.010 24411035

[6] Drake TS Metz SA Grunert GM O'Brien WF. Peritoneal fluid volume in endometriosis. Fertil Steril. (1980) 34:280–1. 10.1016/S0015-0282(16)44963-0 7409252

[7] Donnez J Langerock S Thomas K. Peritoneal fluid volume, 17beta-estradiol and progesterone concentrations in women with endometriosis and/or luteinized unruptured follicle syndrome. Gynecol Obstet Invest. (1983) 16:210–20. 10.1159/000299260 6629142

[8] Sasamoto N Shafrir AL Wallace BM Vitonis AF Fraer CJ Sadler Gallagher J Trends in pelvic pain symptoms over 2 years of follow-up among adolescents and young adults with and without endometriosis. Pain. (2023) 164:613–24. 10.1097/j.pain.0000000000002747 35947080 PMC9908772

[9] Vitonis AF Vincent K Rahmioglu N Fassbender A Buck Louis GM Hummelshoj L World endometriosis research foundation endometriosis phenome and biobanking harmonization project: iI. Clinical and covariate phenotype data collection in endometriosis research. Fertil Steril. (2014) 102:1223–32. 10.1016/j.fertnstert.2014.07.1244 25256930 PMC4252538

[10] Becker CM Laufer MR Stratton P Hummelshoj L Missmer SA Zondervan KT World endometriosis research foundation endometriosis phenome and biobanking harmonisation project: i. Surgical phenotype data collection in endometriosis research. Fertil Steril. (2014) 102:1213–22. 10.1016/j.fertnstert.2014.07.709 25150390 PMC4230690

[11] Rahmioglu N Fassbender A Vitonis AF Tworoger SS Hummelshoj L D'Hooghe TM World endometriosis research foundation endometriosis phenome and biobanking harmonization project: iII. Fluid biospecimen collection, processing, and storage in endometriosis research. Fertil Steril. (2014) 102:1233–43. 10.1016/j.fertnstert.2014.07.1208 25256929 PMC4230639

[12] Use, WHOECoPSt, Interpretation of, A, and World Health, O, Physical status: the use of and interpretation of anthropometry, report of a WHO expert committee, World Health Organization, Geneva, 1995. 8594834

[13] Barlow SE. Expert committee recommendations regarding the prevention, assessment, and treatment of child and adolescent overweight and obesity: summary report. Pediatrics. (2007) 120(Suppl 4):S164–92. 10.1542/peds.2007-2329C 18055651

[14] Horne AW Missmer SA. Pathophysiology, diagnosis, and management of endometriosis. Br Med J. (2022) 379:e070750. 10.1136/bmj-2022-070750 36375827

[15] Maathuis JB Van Look PF Michie EA. Changes in volume, total protein and ovarian steroid concentrations of peritoneal fluid throughout the human menstrual cycle. J Endocrinol. (1978) 76:123–33. 10.1677/joe.0.0760123 624877

[16] Donnez J Nisolle M Casanas-Roux F Brion P Da Costa Ferreira N. Stereometric evaluation of peritoneal endometriosis and endometriotic nodules of the rectovaginal septum. Hum Reprod. (1996) 11:224–8. 10.1093/oxfordjournals.humrep.a019024 8671191

[17] Koninckx PR Kennedy SH Barlow DH. Endometriotic disease: the role of peritoneal fluid. Hum Reprod Update. (1998) 4:741–51. 10.1093/humupd/4.5.741 10027629

[18] Polak G Barczyński B Wertel I Kwaśniewski W Bednarek W Derewianka-Polak M Disrupted iron metabolism in peritoneal fluid may induce oxidative stress in the peritoneal cavity of women with endometriosis. Ann Agric Environ Med. (2018) 25:587–92. 10.26444/aaem/75802 30586986

[19] Donnez J Smoes P Gillerot S Casanas-Roux F Nisolle M. Vascular endothelial growth factor (VEGF) in endometriosis. Hum Reprod. (1998) 13:1686–90. 10.1093/humrep/13.6.1686 9688413

[20] Nisolle M Casanas-Roux F Anaf V Mine JM Donnez J. Morphometric study of the stromal vascularization in peritoneal endometriosis. Fertil Steril. (1993) 59:681–4. 10.1016/S0015-0282(16)55823-3 8458479

