# Supplementary material for: Visualized peritoneal fluid variation in adolescents and young adults with endometriosis: is there more to it?
Source: Front Reprod Health. 2023 Dec 15;5:1297907. doi: 10.3389/frph.2023.1297907 (PMC10757835; doi:10.3389/frph.2023.1297907)
Supplement: Supplementary file 1 [file Table1.docx]

**Supplementary Table 1. Patient characteristics, endometriosis type, lesion location, and color by peritoneal fluid color excluding those who were experiencing vaginal bleeding at surgery or had gross contamination in the peritoneal fluid samples (n=414).**

|  | **Peritoneal fluid color** | | | |  |
| --- | --- | --- | --- | --- | --- |
|  | Yellow (n=108) | Orange (n=35) | Pink (n=37) | Red (n=234) | p-value |
| **Patient and endometriosis-characteristics at time of surgery** | | | | | |
| **Hormone use at time of surgery** |  |  |  |  |  |
| No | 15 (14%) | 8 (23%) | 6 (17%) | 45 (19%) | 0.57 |
| Yes | 92 (86%) | 27 (77%) | 30 (83%) | 188 (81%) |  |
| **Hormone type at time of surgery** |  |  |  |  |  |
| Combined birth control pills | 38 (41%) | 16 (59%) | 18 (60%) | 119 (63%) | 0.05 |
| Progesterone only medication | 37 (40%) | 6 (22%) | 8 (27%) | 49 (26%) |  |
| Progesterone intrauterine devise | 4 (4%) | 2 (7%) | 2 (7%) | 5 (3%) |  |
| Other hormones | 13 (14%) | 3 (11%) | 2 (7%) | 15 (8%) |  |
| **Menstrual cycle phase at the time of surgery among 74 participants not using hormonal therapy** | | | | | |
| Non-cycling (on hormones) | N=92 | N=27 | N=30 | N=188 |  |
| Cycling | N=15 | N=8 | N=6 | N=45 |  |
| Proliferative | 1 (8%) | 0 (0%) | 1 (17%) | 16 (36%) | 0.20 |
| Ovulatory | 3 (23%) | 0 (0%) | 1 (17%) | 7 (16%) |  |
| Secretory | 8 (62%) | 6 (86%) | 3 (50%) | 16 (36%) |  |
| Irregular cycles | 1 (8%) | 1 (14%) | 1 (17%) | 6 (13%) |  |
| **Pain medications within 48 hours before surgery** | | | | | |
| No | 86 (80%) | 33 (94%) | 31 (84%) | 194 (83%) | 0.25 |
| Yes | 22 (20%) | 2 (6%) | 6 (16%) | 40 (17%) |  |
| **Anti-depressants/anti-anxiety medications within 48 hours before surgery** | | | | | |
| No | 83 (77%) | 30 (86%) | 29 (78%) | 196 (84%) | 0.39 |
| Yes | 25 (23%) | 5 (14%) | 8 (22%) | 438 (16%) |  |
| **Endometriosis macro appearance (visualization) on laparoscopy** | | | | | |
| Superficial only | 102 (97%) | 35 (100%) | 35 (95%) | 220 (94%) | 0.24 |
| Endometrioma | 0 (0%) | 0 (0%) | 1 (3%) | 6 (3%) |  |
| Deep | 3 (3%) | 0 (0%) | 0 (0%) | 8 (3%) |  |
| Both endometrioma and deep | 0 (0%) | 0 (0%) | 1 (3%) | 0 (0%) |  |
| **ASRM stage** |  |  |  |  |  |
| Stage I | 84 (81%) | 27 (77%) | 27 (73%) | 179 (76%) | 0.11 |
| Stage II | 20 (19%) | 8 (23%) | 8 (22%) | 39 (17%) |  |
| Stage III | 0 (0%) | 0 (0%) | 1 (3%) | 2 (1%) |  |
| Stage IV | 0 (0%) | 0 (0%) | 1 (3%) | 14 (6%) |  |
| **Location of superficial endometriosis lesions^1^** | | | | | |
| Pelvic Sidewall | 77 (73%) | 28 (80%) | 22 (59%) | 175 (75%) | 0.19 |
| Uterosacral ligament | 18 (17%) | 4 (11%) | 5 (14%) | 37 (16%) | 0.85 |
| Anterior cul-de-sac | 53 (49%) | 17 (49%) | 19 (51%) | 138 (59%) | 0.37 |
| Posterior cul-de-sac | 94 (90%) | 34 (97%) | 35 (95%) | 213 (91%) | 0.55 |
| **Appearance of superficial endometriosis lesions^2^** | | | | | |
| Vascular | 39 (37%) | 16 (46%) | 8 (22%) | 75 (32%) | 0.14 |
| Clear | 99 (94%) | 35 (100%) | 35 (95%) | 224 (96%) | 0.56 |
| Yellow | 3 (3%) | 1 (3%) | 3 (8%) | 16 (7%) | 0.37 |
| Red | 83 (77%) | 26 (74%) | 32 (86%) | 210 (90%) | 0.02 |
| White | 25 (24%) | 10 (29%) | 14 (38%) | 73 (31%) | 0.36 |
| Blue/Black | 17 (16%) | 7 (20%) | 7 (19%) | 68 (29%) | 0.05 |
| Brown | 20 (19%) | 10 (29%) | 10 (27%) | 84 (36%) | 0.02 |
| Any Type A lesions (clear or red) | 101 (94%) | 35 (100%) | 37 (100%) | 230 (98%) | 0.003 |
| Any Type B lesions (brown, blue/black or white) | 48 (44%) | 19 (54%) | 19 (51%) | 150 (64%) | 0.01 |
| **Pain symptoms^3^** |  |  |  |  |  |
| **Dysmenorrhea Severity^4^** |  |  |  |  |  |
| None/Mild | 3 (8%) | 1 (6%) | 1 (6%) | 7 (5%) | 0.93 |
| Moderate | 11 (30%) | 3 (19%) | 6 (33%) | 41 (32%) |  |
| Severe | 23 (62%) | 12 (75%) | 11 (61%) | 82 (63%) |  |
| **Any acyclic pelvic pain^5^** |  |  |  |  |  |
| No | 16 (21%) | 9 (30%) | 6 (24%) | 68 (41%) | 0.02 |
| Yes | 59 (79%) | 21 (70%) | 19 (76%) | 99 (59%) |  |
| **Avoided intercourse or penetration due to dyspareunia^6^** |  |  |  |  |  |
| No | 15 (42%) | 5 (45%) | 1 (14%) | 16 (31%) | 0.40 |
| Yes | 21 (58%) | 6 (55%) | 6 (86%) | 36 (69%) |  |
| **Interrupted intercourse or penetration due to dyspareunia^7^** |  |  |  |  |  |
| No | 14 (39%) | 4 (36%) | 2 (50%) | 17 (35%) | 0.94 |
| Yes | 22 (61%) | 7 (64%) | 2 (50%) | 32 (65%) |  |

Percentages are showing column percentages by the peritoneal fluid colors.

Number of missings for characteristics: hormone use (n=3), hormone type (n=0), menstrual cycle phase among those cycling (n=03, pain medication (n=0), anti-depressants (n=0), endometriosis type (n=3), ASRM stage (n=4).

^1,2^ Percentage of superficial lesion appearance and location present at the time of surgery

^3^ Among n=307 who had answered a questionnaire proximal to their surgery (before or after 6 months)

^4^ Usual severity of pelvic pain with periods among subjects who are cycling (n=209). Number of missing (n=5).

^5^Assessed experience in the last 3 months. Number of missing (n=10).

^6,7^ Assessed in the last 12 months, restricted to (n=110) participants age ≥18 who ever had sexual vaginal intercourse or penetration. Number of missing avoid intercourse (n=4). Number of missing interrupted intercourse (n=10).
